# Fractional Calculus of the Generalized Mittag-Leffler Type Function

**DOI:** 10.1155/2014/907432

**Published:** 2014-08-18

**Authors:** Dinesh Kumar, Sunil Kumar

**Affiliations:** ^1^Department of Mathematics and Statistics, Jai Narain Vyas University, Jodhpur 342005, India; ^2^Department of Mathematics, National Institute of Technology Jamshedpur, Jharkhand 831014, India

## Abstract

We introduce and study a new function called *R*-function, which is an extension of the generalized Mittag-Leffler function. We derive the relations that exist between the *R*-function and Saigo fractional calculus operators. Some results derived by Samko et al. (1993), Kilbas (2005), Kilbas and Saigo (1995), and Sharma and Jain (2009) are special cases of the main results derived in this paper.

## 1. Introduction and Preliminaries

The Mittag-Leffler function has gained importance and popularity during the last one and a half decades due to its direct involvement in the problems of physics, biology, engineering, and applied sciences. Mittag-Leffler function naturally occurs as the solution of fractional order differential equations and fractional order integral equations.

In 1903, the Swedish mathematician Mittag-Leffler [1, 2] introduced the function *E*_*α*_(*z*), defined by

(1) Eα(z)=∑n=0∞znΓ(αn+1), (α∈C,Re(α)>0).

The Mittag-Leffler function *E*_*α*_(*z*) was studied by Wiman [3] who defined the function *E*_*α*,*β*_(*z*) as follows:

(2) Eα,β(z)=∑n=0∞znΓ(αn+β), (α,β∈C,Re(α)>0,Re(β)>0).

The function *E*_*α*,*β*_(*z*) is now known as Wiman function, which was later studied by Agarwal [4] and others.

The generalization of (2) was introduced by Prabhakar [5] in terms of the series representation

(3) Eα,βγ(z)=∑n=0∞(γ)nznΓ(αn+β)n!,(α,β,γ∈C,Re(α)>0,Re(β)>0),

where (*γ*)_*n*_ is Pochhammer's symbol, defined by

(4) (γ)n=Γ(γ+n)Γ(γ)={1,(n=0,γ≠0)γ(γ+1)⋯(γ+n−1),(n∈N,γ∈C).

Shukla and Prajapati [6] defined and investigated the function *E*_*α*,*β*_^*γ*,*q*^(*z*) as

(5) Eα,βγ,q(z)=∑n=0∞(γ)qnznΓ(αn+β)n!, (α,β,γ∈C,Re(α)>0,Re(β)>0,Re(γ)>0),

where *q* ∈ (0,1) ∪ *N* and (*γ*)_*qn*_ = Γ(*γ* + *qn*)/Γ(*γ*) denotes the generalized Pochhammer symbol which in particular reduces to

(6) $$\begin{array}{c} q^{qn}\overset{q}{\underset{r=1}{\prod }}{\left(\frac{\gamma +r-1}{q}\right)}_{n},\;q\in N. \end{array}$$

Srivastava and Tomovski [7] introduced and investigated a further generalization of (3), which is defined in the following way:

(7) Eα,βγ,k(z)=∑n=0∞(γ)knznΓ(αn+β)n!, (z,β,γ∈C;Re(α)>max⁡{0,Re(k)−1};Re(k)>0),

which, in the special case when *k* = *q* (*q* ∈ (0,1) ∪ *N*) and min⁡{*Re*(*β*), *Re*(*γ*)} > 0, is given by (5).

It is an entire function of order *ρ* = [*Re*(*α*)]^−1^. Some special cases of (3) are

(8) Eα(z)=Eα,11(z),  Eα,β(z)=Eα,β1(z),ϕ(β,γ;z)=F11(β,γ;z)=Γ(γ)E1,γβ(z);

here _1_ *F*_1_ denotes an hypergeometric function; see also _2_ *F*_1_ in (10).

**Remark 1 .**

A detailed account of Mittag-Leffler functions and their applications can be found in the monograph by Haubold et al. [8].

An interesting generalization of (2) is recently introduced by Kilbas and Saigo [9] in terms of a special entire function as given below:

(9) $$\begin{array}{c} E_{\alpha ,m,r}\left(z\right)=\overset{\infty }{\underset{n=0}{\sum }}c_{n}z^{n}, \end{array}$$

where *c*_*n*_ = ∏_*i*=0_^*n*−1^(Γ[*α*(*im* + *r*) + 1]/Γ[*α*(*im* + *r* + 1) + 1]) and an empty product is to be interpreted as unity.

### 1.1. Fractional Integrals and Derivatives

An interesting and useful generalization of both the Riemann-Liouville and Erdélyi-Kober fractional integration operators is introduced by Saigo [10] in terms of Gauss's hypergeometric function as follows:

(10) I0+α,β,ηf(z)  =z−α−βΓ(α)∫0z(z−t)α−1F21(α+β,−η;α;1−tz)00000000000000×f(t)dt,

where *α*, *β*, *η* ∈ *C*, *Re*(*α*) > 0, and *z* ∈ *R*_+_, and the generalized fractional derivative of a function

(11) D0+α,β,ηf(z)=zα+βΓ(n−α)(ddx)n ×∫0z(z−t)n−α−1     ×F21(−α−β,n−α−η;n−α;1−tz)f(t)dt,

where *n* = [*Re*(*α*)] + 1.

## 2. The *R*-Function

The *R*-function is introduced by the authors as follows:

(12) Rpkqα,β;γ(z)=Rpkqα,β;γ(a1,...,ap;b1,...,bq;z)=∑n=0∞∏j=1p(aj)n∏j=1q(bj)n(γ)knznn!Γ(αn+β),

where *α*, *β*, *γ* ∈ *C*, *Re*(*α*) > max⁡{0, *Re*(*k*) − 1}; *Re*(*k*) > 0; (*a*_*j*_)_*n*_, and (*b*_*j*_)_*n*_ are the Pochhammer symbols. The series (12) is defined when none of the parameters *b*_*j*_'s, $j=\bar{1,q}$, is a negative integer or zero. If any parameter *a*_*j*_ is a negative integer or zero, then the series (12) terminates to a polynomial in, and the series is convergent for all *z* if *p* < *q* + 1. It can also converge in some cases if we have *p* = *q* + 1 and |*z*| = 1. Let *γ* = ∑_*j*=1_^*p*^ *a*_*j*_ − ∑_*j*=1_^*q*^ *b*_*j*_; it can be shown that if *Re*(*γ*) > 0 and *p* = *q* + 1 the series is absolutely convergent for |*z*| = 1, in order convergent for *z* = −1 when 0 ≤ *Re*(*γ*) < 1 and divergent for |*z*| = 1 when 1 ≤ *Re*(*γ*).

### 2.1. Special Cases of the *R*-Function

(i) If we set *a*_*j*_ = *b*_*j*_ = 1, we have

(13) R0k0α,β;γ(z)=∑n=0∞(γ)knznn!Γ(αn+β)=Eα,βγ,k(z),

where *E*_*α*,*β*_^*γ*,*k*^(*z*) is the generalized Mittag-Leffler function introduced by Srivastava and Tomovski [7]; compare (5).

 (ii) In the special case of (13), when *k* = *q*  (*q* ∈ (0,1) ∪ *N*) and min⁡{*Re*(*β*), *Re*(*γ*)} > 0, we have the following:

(14) R0q0α,β;γ(z)=∑n=0∞(γ)qnznn!Γ(αn+β)=Eα,βγ,q(z),

where *E*_*α*,*β*_^*γ*,*q*^(*z*) was considered earlier by Shukla and Prajapati [6].

 (iii) If we set *a*_*j*_ = *b*_*j*_ = *k* = 1 in (12), we have

(15) R010α,β;γ(z)=∑n=0∞(γ)nznn!Γ(αn+β)=Eα,βγ(z),

where *E*_*α*,*β*_^*γ*^(*z*) is generalization of the Mittag-Leffler function introduced by Prabhakar [5] and studied by Haubold et al. [8] and others; compare (3).

 (iv) If we put *γ* = 1 in (15), we have

(16) R010α,β;1(z)=∑n=0∞znΓ(αn+β)=Eα,β1(z)=Eα,β(z),

where *E*_*α*,*β*_(*z*) is the generalized Mittag-Leffler function [3] (also known as Wiman function), which was later studied by Agarwal [4] and others; compare (2).

 (v) If we take *β* = *γ* = 1 in (15), we have

(17) R010α,1;1(z)=∑n=0∞znΓ(αn+1)=Eα,11(z)=Eα(z),

where *E*_*α*_(*z*) is the Mittag-Leffler function [1, 2]; compare (1).

 (vi) If we take *α* = *β* = *γ* = 1 in (15), we obtain

(18) R0101,1;1(z)=∑n=0∞znΓ(n+1)=E1,11(z)=E1(z)=ex,

where *e* ^*x*^ is the exponential function [11]. 

 (vii) If we set *γ* = *k* = 1 in (12), then the *R*-function can be represented in the Wright generalized hypergeometric function [12] _*p*_ *ψ*_*q*_(*z*) and the *H*-function [13, 14] as given below:

(19) Rp1qα,β;1(z) =Rp1qα,β;1(a1,…,ap;b1,…,bq;z) =Ωψp+1q+1[z|(b1,1),…,(bq,1),(β,α)(a1,1),…,(ap,1),(1,1)] =ΩHp+1,q+21,p+1[−z|(0,1),(1−bj,1)1,q,(1−β,α)(1−aj,1)1,p,(0,1)],hhhhmmhhhmmmmwhere    ∏j=1qΓ(bj)r∏j=1pΓ(aj)r,

where *H*-function is as defined in the monograph by Mathai and Saxena [14]. 

 (viii) If we set *p* = *q* = 0 and *γ* = *k* = 1 in (12), then we obtain another special case of *R*-function in terms of the Wright generalized hypergeometric function as given below:

(20) R010α,β;1(z)=R010α,β;1(−;1;z)=∑n=0∞Γ(n+1)znΓ(αn+β)n!=(1)nznΓ(αn+β)n!=ψ11[z|(β,α)(1,1)].

(ix) If we set *α* = *β* = *γ* = *k* = 1 in (12), then the *R*-function reduces to the generalized hypergeometric function_*p*_ *F*_*q*_ (see for detail [11, 15, 16]) as given below:

(21) Rp1q1,1;1(a1,…,ap;b1,…,bq;z)  =∑n=0∞∏j=1p(aj)n∏j=1q(bj)nznn!=Fpq((aj)1,p;(bj)1,q;z).

## 3. Relations with Generalized Fractional Calculus Operators

In this section we derive two theorems relating to generalized fractional integrals and derivative of the *R*-function.

**Theorem 2 .**

Let *ϑ*, *η*, *δ*, *α*, *β*, *γ* ∈ *C*, *Re*(*ϑ*) > 0, *Re*(*α*) > 0, and (*a*)_*n*_ = Γ(*a* + *n*)/Γ(*a*); then there holds the relation

(22) I0+ϑ,η,δ(Rpkqα,β;γ(x))=x−ηΓ(1+δ−η)Γ(1+ϑ+δ)Γ(1−η) ×Rp+2kq+2α,β;γ(a1,…,ap,1,(1+δ−η);b1,…,bq,mmmmmmmmm(1+ϑ+δ),(1−η);xa1,…,ap,1,(1+δ−η);b1,…,bq).

**Proof**

Following the definition of Saigo fractional integral [17] as given in (10), we have the following relation:

(23) I0+ϑ,η,δ(  pkRqα,β;γ(x)) =x−ϑ−ηΓ(ϑ)∫0x(x−t)ϑ−1F21(ϑ+η,−δ;ϑ;1−tx)  hhhhhh×Rpkqα,β;γ(t) dt;

by virtue of (12), we obtain

(24) I0+ϑ,η,δ(  pkRqα,β;γ(x))  =x−ϑ−ηΓ(ϑ)∫0x(x−t)ϑ−1F21(ϑ+η,−δ;ϑ;1−tx)   hhhhh×∑n=0∞∏j=1p(aj)n∏j=1q(bj)n(γ)kntnn!Γ(αn+β) dt.

Interchanging the order of integration and evaluating the inner integral with the help of Beta function, we arrive at

(25) I0+ϑ,η,δ(Rpkqα,β;γ(x)) =x−ηΓ(1+δ−η)Γ(1+ϑ+δ)Γ(1−η)  ×∑n=0∞∏j=1p(aj)n(1)n(δ−η+1)n∏j=1q(bj)n(ϑ+δ+1)n(1−η)n(γ)knxnn!Γ(αn+β) =x−ηΓ(1+δ−η)Γ(1+ϑ+δ)Γ(1−η)  ×Rp+2 kq+2α,β;γ(a1,…,ap,1,(δ−η+1);b1,…,bq,        (ϑ+δ+1),(1−η);x).

The interchange of the order of summation is permissible under the conditions stated along with the theorem. This shows that a Saigo fractional integral of the *R*-function is again the *R*-function with increased order (*p* + 2, *q* + 2).

This completes the proof of Theorem 2.

If we put *η* = −*ϑ*, then we obtain following Corollary concerning Riemann-Liouville fractional integral operator [16].

**Corollary 3 .**

Let *ϑ*, *α*, *β*, *γ* ∈ *C*, *Re*(*ϑ*) > 0, *Re*(*α*) > 0, and (*a*)_*n*_ = Γ(*a* + *n*)/Γ(*a*), then there holds the relation

(26) I0+ϑ(Rpkqα,β;γ(x)) =xϑΓ(1+ϑ)Rp+1 kq+1α,β;γ(a1,…,ap,1;b1,…,bq,(1+ϑ);x).

**Theorem 4 .**

Let *ϑ*, *η*, *δ*, *α*, *β*, *γ* ∈ *C*, *Re*(*ϑ*) > 0, *Re*(*α*) > 0, and (*a*)_*n*_ = Γ(*a* + *n*)/Γ(*a*), then there holds the relation

(27) D0+ϑ,η,δ(Rpkqα,β;γ(x)) =xηΓ(1+ϑ+η+δ)Γ(1+δ)Γ(1+η)  ×Rp+2 kq+2α,β;γ(a1,…,ap,1,(1+ϑ+η+δ);hhhhhhhhhhh  b1,…,bq,(1+δ),(1+η);x).

**Proof**

Following the definition of Saigo fractional derivative as given in (11), we have the following relation:

(28) D0+ϑ,η,δ(Rpkqα,β;γ(x))  =xϑ+ηΓ(r−ϑ)(ddx)r ×∫0x(x−t)r−ϑ−1F21(−ϑ−η,r−ϑ−δ;r−ϑ;1−tx) mmmm×Rpkqα,β,γ(t) dt,

where *r* = [*Re*(*ϑ*)] + 1.

By virtue of (12), we obtain

(29) D0+ϑ,η,δ(  pkRqα,β;γ(x))=xϑ+ηΓ(r−ϑ)(ddx)r ×∫0x(x−t)r−ϑ−1F21(−ϑ−η,r−ϑ−δ;r−ϑ;1−tx)   ×∑n=0∞∏j=1p(aj)n∏j=1q(bj)n(γ)kntnn!Γ(αn+β) dt.

Interchanging the order of integration and evaluating the inner integral with the help of Beta function, we arrive at

(30) D0+ϑ,η,δ(Rpkqα,β;γ(x))=xr+ηΓ(1+ϑ+η+δ)Γ(1+δ)Γ(1+r+η)∑n=0∞∏j=1p(aj)n(1)n(1+ϑ+η+δ)n∏j=1q(bj)n(1+δ)n(1+r+η)nhhhhhhhhhhhhmmmmmmm×(γ)knxnn!Γ(αn+β)=xr+ηΓ(1+ϑ+η+δ)Γ(1+δ)Γ(1+r+η) ×Rp+2 kq+2α,β;γ(a1,…,ap,1,(1+ϑ+η+δ);b1,…,bq,mmmmmmmmmm(1+δ),(1+r+η);x).

Here *r* = [*Re*(*ϑ*)] + 1, and by using (*d* ^*r*^/*dx* ^*r*^)*x* ^*m*^ = (Γ(*m* + 1)/Γ(*m* − *r* + 1))*x* ^*m*−*r*^, where *m* ≥ *r* in the above expression, we obtain the right-hand side of (27). This shows that a Saigo fractional derivative of the *R*-function is again the *R*-function with increased order (*p* + 2, *q* + 2).

This completes the proof of Theorem 4.

If we put *η* = −*ϑ*, then we obtain following Corollary concerning Riemann-Liouville fractional derivative operator [16].

**Corollary 5 .**

Let *ϑ*, *α*, *β*, *γ* ∈ *C*, *Re*(*ϑ*) > 0, *Re*(*α*) > 0, and (*a*)_*n*_ = Γ(*a* + *n*)/Γ(*a*); then there holds the relation

(31) D0+ϑ(  pkRqα,β;γ(x))=x−ϑΓ(1−ϑ)Rp+1 kq+1α,β;γ(a1,…,ap,1;b1,…,bq,(1−ϑ);x).

**Remark 6 .**

A number of known and new results can be obtained as special cases of Theorems 2 and 4, but we do not mention them here on account of lack of space.

## 4. Conclusion

In this paper we derive a new generalization of Mittag-Leffler function and obtain the relations between the *R*-function and Saigo fractional calculus operators. The results are also extension of work done by Sharma [18]. The provided results are new and have uniqueness identity in the literature. A number of known and new results are special cases of our main findings.
